# Subgingival microbiome of deep and shallow periodontal sites in patients with rheumatoid arthritis: a pilot study

**DOI:** 10.1186/s12903-021-01597-x

**Published:** 2021-05-08

**Authors:** Ryanne Lehenaff, Ryan Tamashiro, Marcelle M. Nascimento, Kyulim Lee, Renita Jenkins, Joan Whitlock, Eric C. Li, Gurjit Sidhu, Susanne Anderson, Ann Progulske-Fox, Michael R. Bubb, Edward K. L. Chan, Gary P. Wang

**Affiliations:** 1 Division of Infectious Diseases and Global Medicine, Department of Medicine, College of Medicine, University of Florida, FL Gainesville, USA; 2 Department of Restorative Dental Sciences, College of Dentistry, University of Florida, Gainesville, FL USA; 3 Department of Oral Biology, College of Dentistry, Center for Molecular Microbiology, University of Florida, Gainesville, FL USA; 4 Dental Clinical Research Unit, College of Dentistry, University of Florida, Gainesville, FL USA; 5 Division of Rheumatology, Department of Medicine, College of Medicine, University of Florida, Gainesville, FL USA; 6 Medical Service, North Florida/South Georgia Veterans Health System, Gainesville, FL USA

**Keywords:** Subgingival microbiome, Rheumatoid arthritis, Microbial dysbiosis, Periodontal disease, 16S rRNA sequencing

## Abstract

**Background:**

Subgingival microbiome in disease-associated subgingival sites is known to be dysbiotic and significantly altered. In patients with rheumatoid arthritis (RA), the extent of dysbiosis in disease- and health-associated subgingival sites is not clear.

**Methods:**

8 RA and 10 non-RA subjects were recruited for this pilot study. All subjects received full oral examination and underwent collection of subgingival plaque samples from both shallow (periodontal health-associated, probing depth ≤ 3mm) and deep subgingival sites (periodontal disease-associated, probing depth ≥ 4 mm). RA subjects also had rheumatological evaluation. Plaque community profiles were analyzed using 16 S rRNA sequencing.

**Results:**

The phylogenetic diversity of microbial communities in both RA and non-RA controls was significantly higher in deep subgingival sites compared to shallow sites (*p* = 0.022), and the overall subgingival microbiome clustered primarily according to probing depth (i.e. shallow versus deep sites), and not separated by RA status. While a large number of differentially abundant taxa and gene functions was observed between deep and shallow sites as expected in non-RA controls, we found very few differentially abundant taxa and gene functions between deep and shallow sites in RA subjects. In addition, compared to non-RA controls, the UniFrac distances between deep and shallow sites in RA subjects were smaller, suggesting increased similarity between deep and shallow subgingival microbiome in RA. *Streptococcus parasanguinis* and *Actinomyces meyeri* were overabundant in RA subjects, while *Gemella morbillorum*, *Kingella denitrificans*, *Prevotella melaninogenica* and *Leptotrichia *spp. were more abundant in non-RA subjects.

**Conclusions:**

The aggregate subgingival microbiome was not significantly different between individuals with and without rheumatoid arthritis. Although the differences in the overall subgingival microbiome was driven primarily by probing depth, in contrast to the substantial microbiome differences typically seen between deep and shallow sites in non-RA patients, the microbiome of deep and shallow sites in RA patients were more similar to each other. These results suggest that factors associated with RA may modulate the ecology of subgingival microbiome and its relationship to periodontal disease, the basis of which remains unknown but warrants further investigation.

**Supplementary Information:**

The online version contains supplementary material available at 10.1186/s12903-021-01597-x.

## Background

The human microbiome is a complex collection of microbial communities inhabiting many sites in the human body that perform functions required for the health of a host. The interactions between our immune system and the microbiome generally mediate a symbiotic relationship, where pathogenic bacteria are reduced, and commensal or beneficial organisms are tolerated. Many studies have shown that the microbiome plays a crucial role in the training and development of our immune system [1]. For example, intestinal epithelial cells of neonates require early exposure to bacterial endotoxin in order to establish mucosal homeostasis and subsequently, permit microbial colonization without sustaining damage [2]. As a result, there has been an increased interest in exploring the role of microbiome in autoimmune diseases. Indeed, microbial dysbiosis has been associated with several diseases characterized by immune dysregulation, including systemic lupus erythematosus, inflammatory bowel disease, and multiple sclerosis, among others [3]. Compared to these autoimmune conditions, the role of the human microbiome in rheumatoid arthritis (RA) has received little attention.

RA is an autoimmune disease marked by chronic joint inflammation and pain, which can progress to irreparable cartilage and bone damage [4]. The etiology of RA remains unknown, but both genetic predispositions and environmental triggers are likely responsible [5]. Dysbiosis of the subgingival microbiome and the resulting periodontitis have been implicated as an environmental trigger [5–7]. Periodontal disease, a condition often associated with changes in the subgingival microbiome, is a multifactorial disease marked by inflammation and destruction of tooth-supporting tissue [8]. Individuals with RA have an increased incidence and severity of periodontal disease as compared to their healthy counterparts [9, 10]. Even after controlling for arthritic symptoms, patients with RA are more likely to have chronic periodontitis [9–13]. Furthermore, the pathophysiology of RA and chronic periodontitis shares similar features, both involving immune cell infiltration into tissue, initiation of an inflammatory environment, and subsequent tissue destruction [4, 14]. Given these shared characteristics, the relationship between rheumatoid arthritis and periodontal disease is an active area of investigation.

Several studies have examined the associations between specific subgingival microbes and RA symptoms. [9, 15–18]. Elevated levels of DNA from *Porphyromonas gingivalis*, a pathogen commonly associated with periodontal disease, have been found in the synovial fluid of RA patients [19]. In vitro studies have shown that *P. gingivalis* is capable of infiltrating primary chondrocytes and causing cell damage [20, 21]. In addition, *P. gingivalis* expresses peptidylarginine deiminase and produces citrullinated human proteins, which are key molecules in the initiation and progression of RA. [22]. Similarly, *Aggregatibacter actinomycetemcomitans* produces leukotoxin A, which increases the production and release of citrullinated proteins in neutrophils. Indeed, RA patients have higher rates of leukotoxin A exposure compared to their healthy counterparts [23]. This exposure to citrullinated proteins results in the development of autoantibodies to citrullinated proteins (ACPAs) in genetically susceptible individuals [24]. Taken together, these studies suggest that oral bacteria or their metabolites may translocate from the mouth to the joints, infiltrate chondrocytes, increase citrullinated proteins, and cause cellular damage, potentially contributing to the pathogenesis of RA.

In limiting the analysis to a few selected organisms, most prior studies did not account for the complexity and dynamics of the overall subgingival microbiome, which collectively may influence RA pathogenesis. Recent advances in sequencing technology have provided a greater resolution of the subgingival microbiome, allowing for both species- and community-level comparisons between individuals. A few studies have shown that the salivary microbiome differs between healthy and RA patients [25, 26], but data for subgingival microbial dysbiosis have been mixed [15–18]. In most studies, the subgingival microbiome of subjects with and without RA share similar diversity [15, 18]. In addition, the influence of RA disease severity on microbial diversity remains unclear as increased disease activity was both negatively [17] and positively [15] associated with different diversity metrics. Lopez-Oliva et al. [16] found that RA influenced the overall structure of subgingival communities, but this was not supported by other studies [15, 17, 18].

Given the similarities in pathophysiology, shared risk factors, and the increased incidence and severity of periodontal disease in RA subjects, there is significant interest in investigating the linkage between these two disease processes. Previous studies of subgingival microbiome in RA patients have shown conflicting results. Several studies did not account for the severity of periodontal disease in their sampling process, as only the deepest subgingival sites or random sites were sampled [15–17]. In this pilot study, we analzyed the subgingival microbiome of both shallow (health-associated) and deep (disease-associated) subgingival sites in patients with rheumatoid arthritis (RA), and then compare to non-RA household controls. We hypothesized that subgingival microbiome of diseased and healthy subgingival sites in RA patients are altered compared to non-RA controls, which may offer insights into the interplay between subgingival microbiome and RA pathogenesis.

## Methods

### Study population

A total of 10 subjects with rheumatoid arthritis (RA) who were naïve to biologic therapy and 10 household members of the RA patients (non-RA controls) were recruited from the Rheumatology Clinic at UF Health, Gainesville, Florida. Subjects were excluded if they were (1) younger than 18 years of age, (2) had fewer than 8 natural teeth, (3) antibiotic treatment in the three months preceding sample collection, (4) immune compromising conditions (i.e., patients with HIV/AIDS, or on immunosuppressive regimens for conditions other than rheumatoid arthritis), or (5) participation in another clinical study involving the use of dental products one week prior to sample collection or during the study period. Informed consent was obtained from all subjects. The protocol was approved by the Institutional Review Board at the University of Florida. All RA subjects were on DMARDs at the time of sampling, and 75% were in remission. No RA subjects were on glucocorticoids or had been previously treated with biologics (e.g. adalimumab, etanercept, or golimumab).

### Medical and oral examinations

Each RA patient underwent extensive medical screening including evaluation of autoantibodies (RF and ACPA) and inflammatory markers (ESR and CRP). Additionally, RAPID3 score, a measure of RA severity, was determined and the treatment history of RA was collected. Two of the 10 RA patients were excluded from the final analysis, as one subject had previously received biologic therapy and the diagnosis of a second subject was subsequently felt to be more consistent with mixed connective tissue disease rather than RA. Of the 8 RA subjects included in the study, 7 were seropositive for RA. Determination of remission was based on ACR/European League Against Rheumatism (EULAR) 2011 remission criteria [27]. Information on previous and recent dental treatments were not collected during the study visit.

 All study participants underwent oral examination and collection of subgingival plaque samples in the Dental Clinical Research Unit at UF Health. Oral examinations included the assessment of dental and periodontal health status by the same dentist (MMN). Subjects were grouped according to periodontal health status as presenting periodontal health or periodontitis [8]. Periodontal health was defined as presenting sites with both probing depth (PD) and clinical attachment level (CAL) ≤ 3 mm, and bleeding on probing (BoP) < 10 %. Periodontitis at different stages of severity (I to III) was defined as presenting: (1) more than 30 % of the tooth sites with both PD and clinical attachment level (CAL) ≥ 4 mm and BoP, and (2) minimum of 6 teeth with at least one site of PD and CAL ≥ 5 mm and BoP as described elsewhere [8, 28].

### Sample collection

Subjects were required to refrain from tooth brushing, flossing, eating, and drinking (anything other than water) for 12 h prior to sample collection. For each subject, one subgingival plaque sample was obtained from a shallow site (probing depth ≤ 3mm) and a second sample from a deep subgingival site (periodontal disease-associated, probing depth ≥ 4 mm).

Each type of plaque sample was pooled from 1 to 6 sites on a single tooth or multiple teeth with similar health conditions using sterile periodontal paper points and transferred directly to a microcentrifuge bead-beating tube containing buffer for storage (PowerBead tubes, Qiagen, Venlo, Netherlands). Sample tubes were placed on ice, and stored at − 80 °C until processing.

### Microbial profiling and microbiome analysis

Bacterial DNA was extracted using the DNeasy Powersoil Kit (Qiagen, Venlo, Netherlands) with the following modifications. An SDS-containing solution was added to the PowerBead tubes to facilitate cellular lysis and membrane breakdown. Tubes were secured to a Vortex Adapter, mixed for 10 min and heated/mixed at 70 °C for 10 min. The supernatant was transferred to a clean, 2 mL collection tube and a solution which precipitates non-DNA material was added. Samples were incubated at 4 °C for 10 min, centrifuged and the supernatant was transferred to another clean tube. This step was repeated, and then a highly concentrated salt solution was added to facilitate DNA binding to the spin column. Samples were then loaded onto spin columns and washed twice to remove contaminants. Purified DNA was eluted from the column and stored at − 20 °C until further processing.

The 16S rRNA genes (V1-V3 region) were amplified using primers 27F (5′-AGAGTTTGATCCTGGCTCAG-3′) and 534R (5′-ATTACCGCGGCTGCTGG-3′) with barcodes to allow multiplex deep sequencing as described previously [29]. The final PCR reactions contained 0.75 U Accuprime Taq High Fidelity Polymerase (Invitrogen, Carlsbad, CA), 2 µL 10X PCR buffer II, 100 nM forward primer, 100 nM reverse primer, 2 µL purified DNA template and sterile DNA/RNA-free water in a total volume of 20 µL. Cycling conditions were as follows: denaturation at 95 °C for 2 min, 25 cycles of denaturation at 95 °C for 20 s, annealing at 56 °C for 30 s and extension at 72 °C for 5 min. PCR products for each sample were analyzed on a SYBR Safe 1 % agarose gel (Invitrogen, Carlsbad, CA) to confirm an expected size of 600 base pairs. The amplicons were excised, purified using NucleoSpin Gel and PCR Clean-up kit (Macherey-Nagel, Bethehem, PA), and then quantified using Qubit HS DNA quantification kits (Invitrogen, Carlsbad, CA). Equimolar concentrations of DNA were then pooled and purified, and qPCR was performed using the Library Quant Kit (Kapa Biosystems, Wilmington, MA). 16 S rRNA sequencing was performed on the Illumina MiSeq platform using the MiSeq Reagent kit V3 and PhiX control V3 kit (Illumina, San Diego, CA).

Raw paired-end MiSeq reads of 300 nucleotides each (covering the V1–V3 hypervariable region of the 16 S rRNA gene using primers 27 F and 534R) were processed using custom scripts in R v.3.4.2 (R Core Team, 2018). Reads were filtered based on exact matches to barcode/primer and an average quality score of 30. Samples were de-multiplexed according to the combination of their unique variable length barcodes (4 to 8 nucleotides) on each paired end. The barcodes and primers were trimmed for downstream analysis. To reconstruct the original contiguous amplicon, paired-end reads were joined using FLASh (Fast Length Adjustment of SHort reads), with a minimum overlap of 10 base pairs. USEARCH alignment was employed with a 97% identity and 80% aligned query threshold to assign OTUs with reference taxonomic information from the Human Oral Microbiome Database (16 S rRNA RefSeq Version 10.1) [30, 31] to each joined read. Reads that did not meet the filtering criteria were excluded from subsequent analysis. Alpha and beta diversity metrics were generated in QIIME2 (version 2018.8, available at https://qiime2.org/) [32] using the core-metrics-phylogenetic pipeline and later analyzed in R v.3.4.2 (R Core Team, 2018). Reads for each sample were rarefied to 7500 prior to alpha and beta diversity analysis.

### Statistical analysis

Age, ESR, RAPID3 and probing depth of periodontal sites (deep vs. shallow) were compared between RA and non-RA controls using Student’s t-tests. The other demographic and clinical were compared using Fisher’s Exact test. All statistical analyses were conducted in R v.3.4.2 (R Core Team, 2018) unless otherwise stated.

For microbiome analysis, mixed linear models were conducted using lmer() in the lme4 package (v.1.1–19) [33] to determine how RA status, probing depth, and their interaction influenced each alpha diversity metric. Subject identity was included as a random effect to account for the non-independence of the deep and shallow samples coming from the same subject. Within RA patients, mixed linear models were used to determine how alpha diversity varied with RA disease activity, measured using the RAPID3 score. Again, subject identity was included as a random effect. To compare the overall community composition, permutational multivariate analysis of variance (PERMANOVA) was performed on weighted and unweighted UniFrac distances between samples using adonis() in vegan v.2.5-3 [34]. UniFrac distances are distance metrics that incorporate phylogenetic relatedness and account for the presence (unweighted) or abundance (weighted) of OTUs. Clustering was visualized with Principal Coordinate Analysis using betadisper() in vegan v.2.5-3 [34]. Differential abundance analysis was performed using LEfSe [35] to identify OTUs associated with RA status or probing depth. Differentially abundant features met the minimum LDA threshold of 2. To compare how the community differences between shallow and deep sites of a single individual differed across RA status, intra-subject distances (the UniFrac distance between shallow and deep site of the same subject) were tested using unpaired t-tests, as subjects could not be paired with their household match because two RA subjects were excluded and not all RA patients had both a shallow and a deep site. Metagenomic functions were predicted using PICRUSt [36] and subjected to biomarker analysis using LEfSe. Differentially abundant gene functions met a minimum LDA threshold of 2. The bioBakery Python tools package contained the Python scripts used in LEfSe and PICRUSt analyses.

 All methods were carried out in accordance relevant guidelines and regulations.

## Results

Clinical characteristics and demographics of RA and non-RA controls are shown in Table 1. No significant differences in age, gender, race, number of caries, and periodontal health status were observed between the two groups.

**Table 1:** Demographics and clinical characteristics of RA and control subjects

Characteristic	Biologic-naïve RA (n = 8)	Non-RA control (n = 10)	*P*-value*
Age, years, mean	55.1 (16.5)	53.2 (16.0)	0.81
Female, %	62.5%	70.0%	1.0
Race^+^, %			
Caucasian Non-Caucasian African American Hispanic Indian Unknown	50% 50% 25% 12.5% 12.5% 0%	30% 70% 10% 10% 0% 50%	0.63
BMI (kg/m^2^)	32.6	–	
Diabetes mellitus	0%	–	
Smoking history			
Current	12.5%	–	
Former	37.5%	–	
Never	50%	–	
RA disease characteristics			
Remission, %	75%	–	–
Autoantibody status			
RF positive, %	75%	–	–
ACPA positive, %	75%	–	–
ESR mm/h, mean	23.38 (17.73)	–	–
CRP mg/L, mean	4.26 (1.64)	–	–
RAPID3 score, mean	10.42 (5.71)	–	–
Medication history			
DMARDs, %	100%	–	–
Glucocorticoids, %	0%	–	–
Biologic agents			
TNF-inhibitor, %	0%	–	–
Other, %	0%	–	–
Number of caries, mean	3 (3.85)	4.4 (7.07)	0.62
Probing depth, mean (mm)			
Shallow sites (PD ≤ 3 mm)	2.58	2.08	0.02
Deep sites (PD ≥ 4 mm) ^$^	4.00	3.65	0.47
Global PD status			
Healthy, %	12.5%	0%	0.54
Periodontitis stage I, %	37.5%	40%	
Periodontitis stage II, %	12.5%	40%	
Periodontitis stage III, %	37.5%	20%	

RF, rheumatoid factor; ACPA, anti-cyclic citrullinated protein antibody; ESR, erythrocyte sedimentation rate; CRP, C-reactive protein; RAPID3, routine assessment of patient index data 3; DMARD, disease modifying antirheumatic drug; TNF, tumor necrosis factor; PD, periodontal disease. Periodontitis stages are described elsewhere [8, 28]

*Age and pocket depth were compared using Student’s t-test. All other parameters were compared using Fisher’s Exact test. P-value in race was calculated with Caucasian vs. non-Caucasian data. Standard deviation is shown in parentheses

^+^Race was self-reported

^$^Probing depths were averaged measurements from the facial and/or lingual sites from which subgingival samples were collected

A total of 34 samples were collected, including both shallow and deep sites from each of the 10 non-RA controls and 6 RA subjects. For the remaining 2 RA subjects, a deep site was not collected from one (G1-10) and a second had no deep sites to sample (G1-2). 16 S rRNA Illumina sequencing of the 34 samples generated 970,260 raw reads. After trimming and quality control, 882,526 reads were available for analysis with an average of 25,957 reads per sample (range: 8246−50,936 reads). Reads clustered into 499 distinct operational taxonomic units (OTUs), belonging to 10 different phyla and 97 different genera. The most abundant phyla were *Firmicutes* (29.9%), *Bacteroidetes* (27.1%), *Fusobacteria* (21.3%) and *Proteobacteria* (13.2%). The dominant genera included *Fusobacterium* (17.9%), *Prevotella* (14.6%), *Veillonella* (11.0%), *Streptococcus* (10.1%), *and Porphyromonas* (7.1%).

**Fig. 1:**
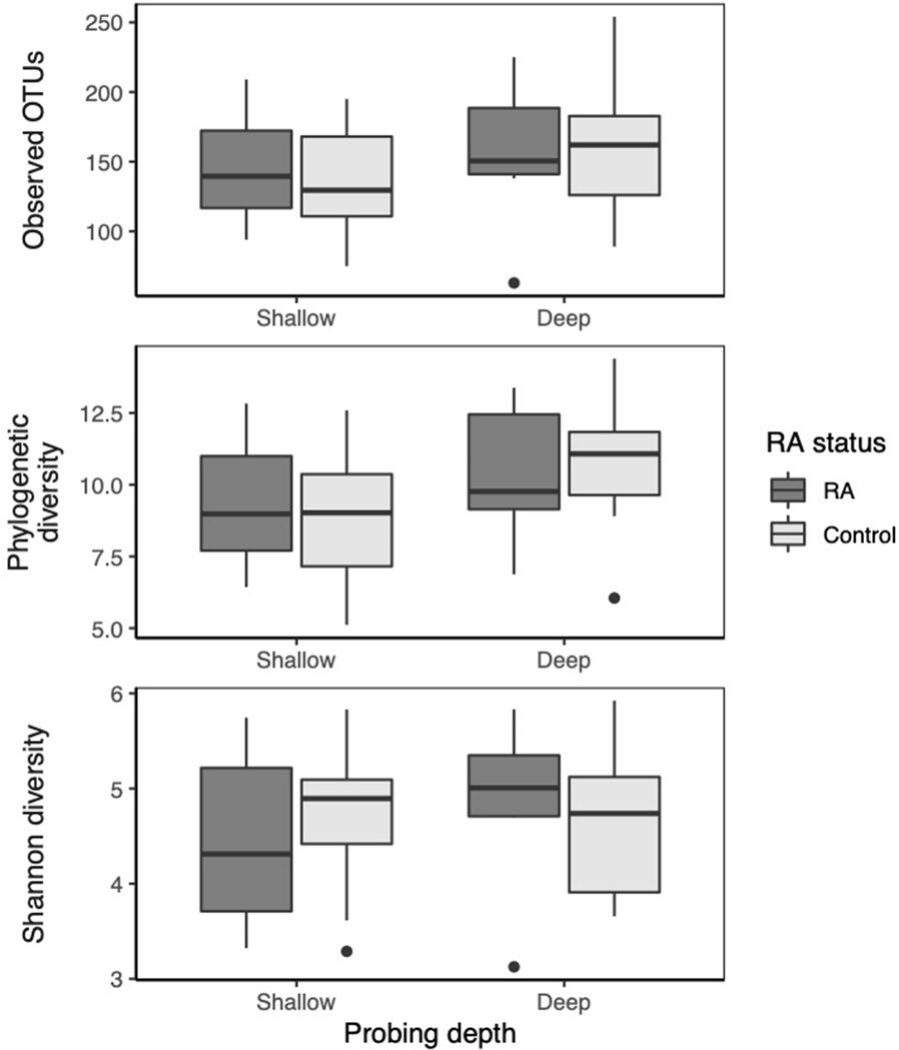
Differences in alpha diversity between RA status and probing depth. Alpha diversity was estimated using three different metrics: observed OTUs, Faith’s phylogenetic distance, and Shannon diversity. Boxplots show the median value and interquartile range (IQR) for each metric. Whiskers extend up to 1.5 times the IQR, and outliers that fall outside of that range are shown as dark points. Statistical significance of differences between groups is shown in Table 2. *OTUs* operational taxonomic units

To characterize the subgingival microbiome in RA, we first calculated the alpha diversity using observed OTUs, Faith’s phylogenetic diversity index, and Shannon’s diversity index (Fig. 1). Analysis using mixed linear models showed that microbial communities in deep subgingival sites had higher phylogenetic diversity than shallow sites (b = 1.93 ± 0.73SE, *p* = 0.022; Table 2). However, neither RA status (b = 0.60 ± 1.10SE, *p* = 0.69) nor the interaction between RA and probing depth (i.e. deep versus shallow sites) was associated with phylogenetic diversity (b = − 1.08 ± 1.20SE, *p* = 0.38). Furthermore, neither RA status nor probing depth was associated with observed OTUs or Shannon diversity. Mixed linear models showed that RA disease activity, as measured by the RAPID3 score, was not associated with differences in any of the alpha diversity measurements for both shallow and deep sites (see Additional file 2: Table S1).

**Table 2:** Effect of probing depth and RA on alpha diversity of subgingival microbiome

	Observed OTUs	Faith’s phylogenetic diversity	Shannon diversity
Probing depth (ref: shallow)	26.50 ± 14.41SE, *p* = 0.087	1.93 ± 0.73SE, *p* = 0.022	− 0.09 ± 0.28SE, *p* = 0.749
RA status (ref: control)	8.45 ± 22.01SE, *p* = 0.704	0.60 ± 1.10SE, *p* = 0.688	− 0.28 ± 0.40SE, *p* = 0.482
Probe depth × RA status	− 18.20 ± 23.08SE, *p* = 0.443	− 1.08 ± 1.20SE, *p* = 0.384	0.52 ± 0.45SE, *p* = 0.275

Mixed linear models were used to examine the influence of probing depth (deep vs. shallow), RA status, and the interaction of the two conditions on three different alpha diversity metrics: observed OTUs, Faith’s phylogenetic diversity, and Shannon diversity. Subject identity was included as a random effect. Model coefficient estimates, the standard error (SE) of the estimates, and p values are shown. OTUs: operational taxonomic units

**Fig. 2:**
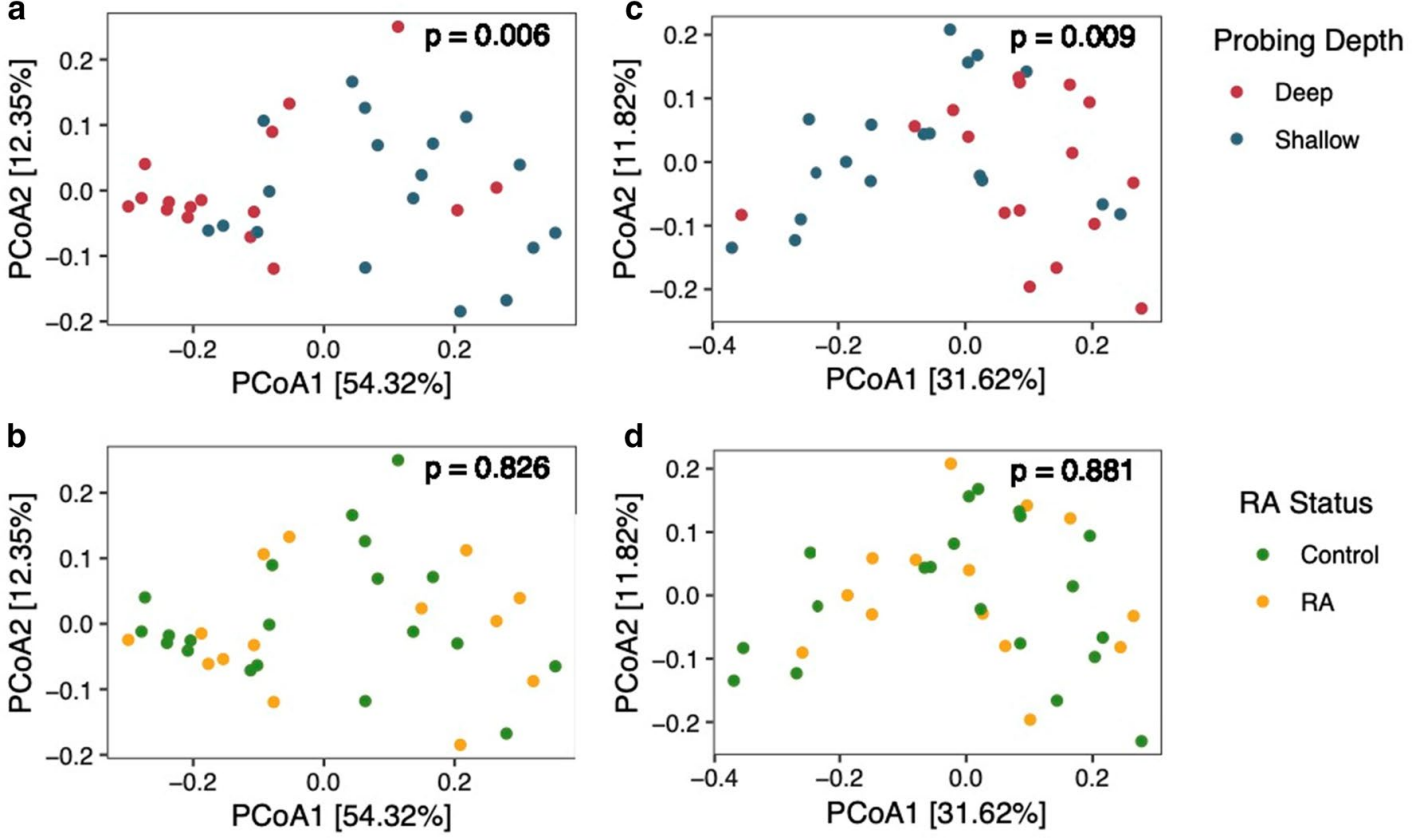
Comparison of subgingival microbiome according to probing depth and RA status. Principal Coordinates Analysis (PCoA) was conducted using weighted and unweighted UniFrac distances. PCoA on weighted UniFrac distances were used to examine community structure according to **a** probing depth and **b** RA status. PCoA on unweighted UniFrac analysis were used to visualize the relationship between **c** probing depth or **d** RA status and community membership. Variation explained by each axis is shown in brackets. lues were estimated using Permutatinal Multivariate Analysis of Variance (PERMANOVA) with 999 permutations

Next, we compared differences at the microbial community level by calculating weighted and unweighted UniFrac distances between samples (Fig. 2). For both weighted (Fig. 2a, b) and unweighted (Fig. 2c, d) UniFrac analysis, samples clustered primarily by probing depth along the first principal coordinate axis (PERMANOVA, weighted UniFrac: *p* < 0.006; unweighted UniFrac: *p* < 0.009). In contrast, samples did not separate according to RA status (PERMANOVA, weighted UniFrac: *p* = 0.826; unweighted UniFrac: *p* = 0.881). Additionally, we found no interaction between probing depth and RA status (PERMANOVA, weighted UniFrac: *p* = 0.861; unweighted UniFrac: *p* = 0.997).

**Fig. 3:**
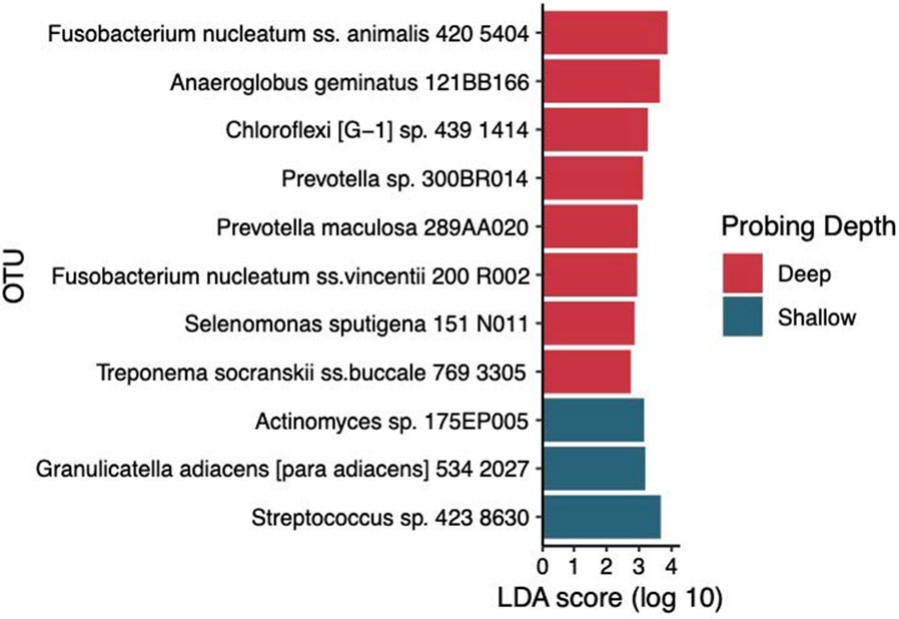
Differentially abundant OTUs between deep and shallow sites in RA subjects. Differentially abundant taxa were identified using LEfSe and met the minimum LDA score of 2. OTUs enriched in deep sites are shown in red, whereas OTUs more abundant in shallow sites are shown in dark blue. OTU: Operational taxonomic unit

**Fig. 4:**
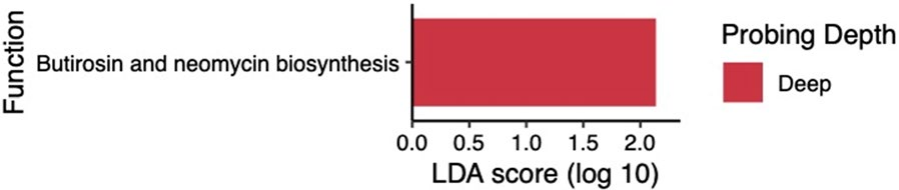
Differentially abundant metagenome functions between deep and shallow sites in RA subjects. Gene functions were predicted using 16 S rRNA data and PICRUSt. Differentially abundant genes were identified using LEfSe and met the minimum LDA score of 2. Gene functions enriched in deep sites are shown in red. There were no gene functions associated with shallow sites

To further compare microbial composition, we utilized Linear discriminant analysis Effect Size (LEfSe) to identify specific OTUs that were differentially abundant between groups. A total of 45 OTUs were differentially enriched between deep and shallow sites in non-RA controls (see Additional file 1: Fig. S1). OTUs abundant in deep subgingival sites of non-RA subjects included several members of the red and orange complexes [37], and were associated with 33 differentially enriched metagenome functions predicted by PICRUSt (see Additional file 1: Fig. S2). Surprisingly, only 11 OTUs (see Fig. 3) and one predicted metagenome function (see Fig. 4) in RA subjects were differentially abundant between deep and shallow sites. Thus, in contrast to the substantial differences in subgingival microbiome between shallow and deep sites in non-RA controls, these results suggest increased similarity in the microbiome of shallow and deep periodontal sites in RA patients.

**Fig. 5:**
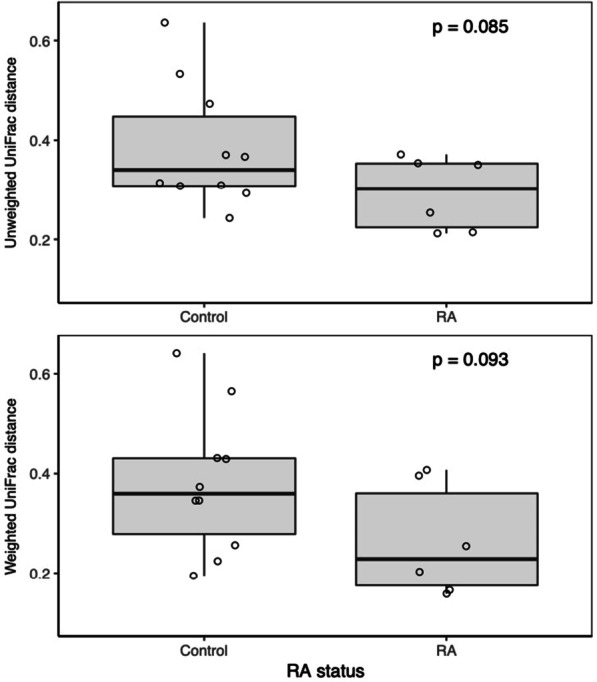
Similiarities between shallow and deep subgingival microbiome within subjects in RA and non-RA controls. **a** Weighted and **b** unweighted UniFrac distances between paired deep and shallow samples were calculated for each subject and then compared between RA and non-RA controls. The smaller the intra-subject UniFrac distance (y-axis), the more similar the paired samples are to each another. Each subject is shown as a single open circle. Boxplots show the median distance and their interquartile range (IQR). Whiskers extend to 1.5 times the IQR. P values were estimated using unpaired t-tests

To quantify these differences, we calculated the weighted and unweighted Unifrac distances between paired sites (deep versus shallow) within each subject, then compared between RA and non-RA controls. Using this approach, large intra-subject distances indicate a high degree of separation between shallow and deep site microbiomes, whereas small distances suggest more similiar microbiomes. The weighted and unweighted intra-subject UniFrac distances for RA patients were 31 and 24% lower than non-RA controls, respectively (Fig. 5), although these differences did not achieve statistical significance (*p* = 0.093 and *p* = 0.085, respectively, unpaired t-tests). Thus, these results support a trend toward increased similarity in the subgingival microbiome between shallow and deep sites in RA.

**Fig. 6:**
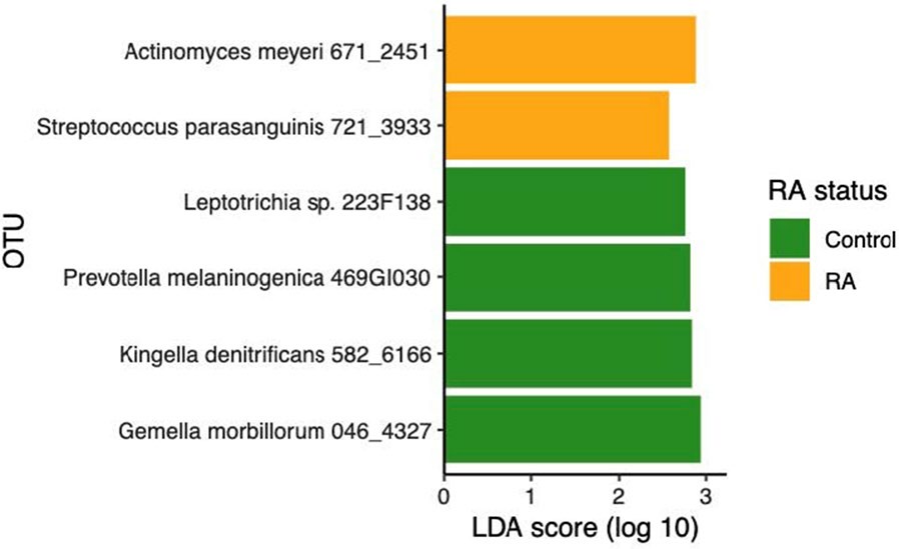
Differentially abundant OTUs in RA and non-RA controls. Differentially abundant OTUs were identified by LEfSe with a minimum LDA threshold of 2. Taxa enriched in non-RA controls are indicated by green bars. Those enriched in RA subjects are indicated by yellow bars. The OTUs associated with RA or non-RA controls shown were observed for both shallow and deep sites. OTU, Operational taxonomic unit

Finally, we examined whether there were specific OTUs in the subgingival microbiome that were unique to RA subjects. Differentially abundant OTUs between RA and non-RA controls were identified using Linear discriminant analysis Effect Size (LEfSe). For both shallow and deep sites, *Actinomyces meyeri* and *Streptococcus parasanguinis* were more abundant in RA subjects compared to non-RA controls, whereas *Gemella morbillorum*, *Kingella denitrificans*, *Prevotella melaninogenica*, and a *Leptotrichia* species were depleted in RA subjects (Fig. 6). *G. morbillorum* was the most abundant OTU among the 6 differentially enriched OTUs but only constituted 0.31% of the reads in non-RA controls and 0.26% in RA, indicating that the four differentially abundant OTUs associated with RA were all minority components of the subgingival microbiome. There were no differences in the abundance of predicted gene functions between RA and non-RA controls.

## Discussion

In this pilot study, the subgingival microbiome of shallow and deep periodontal sites in patients with rheumatoid arthritis (RA) was determined by 16 S rRNA sequencing and compared to those of non-RA household controls. Historically, methods such as organism-specific PCR/qPCR and anaerobic culture techniques have been used to examine subgingival communities in RA patients [38–40]. However, these methods could not compare the overall microbial community profile. Only in the past decade have rapid advances in deep-sequencing technology allowed for an unbiased and more complete picture of the oral microbiome. Prior studies have used Illumina 16 s rRNA sequencing, Roche/454 16 S rRNA sequencing, or whole-genome shotgun sequencing to characterize the oral microbiome in RA [15–18, 25, 26]. Rather than sampling only the deepest subgingival sites as previously reported [17], we sampled paired shallow and deep sites of each subject to define the relationship between healthy and diseased subgingival microbiome within individuals in the context of RA.

It has been reported that periodontal disease is more severe and more prevalent in patients with rheumatoid arthritis [9]. Thus it was not surprising to observe here greater pocket depths for both clinically healthy and diseased sites in RA than the controls. Consistent with previous work [41], we found an increase in phylogenetic diversity for deep sites compared to shallow sites, suggesting that the dysbiosis seen in periodontal disease may be characterized by increased microbial diversity. This is a very interesting finding because it differs from a general trend observed in other body habitats where dysbiosis and disease progression often involve a loss of microbial diversity [42, 43].

Consistent with previous studies [15, 17, 18] with the exception of Ganesan et al. [44] and Dabdoub et al. [45], we observed clustering of subgingival microbiomes by probing depth in both weighted and unweighted UniFrac analysis. However, we did not observe clustering according to RA status. This is consistent with some studies [15, 18] but is in contrast with others [16, 26]. The reasons for these discrepancies are unclear but may be related to the chronicity and/or remission status of RA patients, severity of periodontal disease, use of controls within the family unit, the number of sampling sites, and/or sequencing methods.

Ganesan et al. and Dabdoub et al. found large differences in subgingival communities between subjects with periodontal disease and those who are periodontally healthy [44, 45]. However, their work did not find differences between shallow and deep sites within the same subjects, suggesting that microbial dysbiosis is a global event rather than a local shift [44, 45]. Here, we found that even in the same individual, the communities of shallow and deep sites are different, implying that dysbiosis is a local event. Interestingly, the analysis of intra-subject UniFrac distances and LEfSe suggest that the differences in subgingival microbiome between deep and shallow sites were smaller in RA patients compared to non-RA controls. One possible explanation for the increased similarity in microbiome between shallow and deep sites of RA patients is that subgingival dysbiosis may have already begun in shallow sites, thereby reducing the microbial community differences between shallow and deep sites in RA. Given the small sample size of our pilot study, further investigations using longitudinal sampling and a larger study cohort are needed to confirm these findings.

The analysis of microbiota composition revealed that two OTUs, *S. parasanguinis* (OTU 721) and *A. meyeri* (OTU 671), were more abundant in RA as compared to controls. While neither OTU has been associated with RA to date, several *Actinomyces* species have been shown to be enriched [16] and several *Streptococcus* species were depleted in RA patients [16, 18]. *S. parasanguinis* is an oral commensal capable of bacterial antagonism, inhibiting the growth of periodontal pathogens including *Aggregatibacter actinomycetemcomitans*, *P. gingivalis* and *Prevotella intermedia* [46]. However, one study showed that *S. parasanguinis* contributed to *P. gingivalis* colonization [47]. Nonetheless, this organism has been associated with refractory periodontitis [48] and alveolar bone loss [49], and its role in the colonization of periodontal pathogens may be variable. *A. meyeri* is an opportunistic pathogen and has been shown to cause empyema necessitans [50]. The role of *A. meyeri* in periodontal disease has not been well studied, though this organism has been associated with apical periodontitis [51]. *P. gingivalis* possesses mechanisms for citrullinated proteins, a significant factor in the initiation and propagation of RA [22]. However, we did not find an association with RA in our study.

We identified 4 OTUs underrepresented in RA subjects: *G. morbillorum* (OTU 046), *K. denitrificans* (OTU 582), *P. melaninogenica* (OTU 469) and *Leptotrichia* spp. (OTU 223). *G. morbillorum* has been associated with oral infections, including endodontic disease [52], apical periodontitis [53], and chronic periodontitis [54, 55]. This organism has not been directly associated with RA previously, but it has been reported to cause septic arthritis [56], including in RA patients [57]. Similarly to *G. morbillorum*, *K. denitrificans* is an oral and respiratory tract commensal which can cause infections such as endocarditis and peritonitis in susceptible individuals [58, 59]. This OTU was also depleted in salivary samples of RA patients compared to controls [25]. Contrary to our findings, enrichment of both *Prevotella* and *Leptotrichia* species has been shown in RA [16, 18]. Increase abundance of *P. melaninogenica* in the subgingival microbiome has been associated with a number of different conditions, including inflammatory bowel disease [60], obesity [61], and rheumatoid arthritis and osteoarthritis patients [26]. Furthermore, elevated levels of antibodies to *P. melaninogenica* have been reported for RA patients [62], thus implicating a role in RA [63]. Given the differences between our data and previous work, the role of *P. melaninogenica* and *Leptotrichia* species in RA pathogenesis warrants further investigation.

There are several limitations in this study. First, although the probing depths for deep sites were comparable between RA and non-RA controls, the shallow sites (probing depth ≤ 3 mm) were deeper in RA patients than non-RA controls. These differences in probing depths, however, did not contribute to differences in the microbiome structure of shallow sites between RA and non-RA controls. Second, we did not collect information on smoking history or detailed medical histories, both of which could have influenced the composition of subgingival microbiome. Third, while none of the RA patients received biologic therapy, they were not all receiving the same drug regimens for RA which could have contributed to variations in the microbiome. Lastly, given that this was a pilot study, our sample size was small. Nonetheless, our data suggest that RA may modulate the subgingival microbiome and support further investigation in a larger cohort study to confirm these results.

## Conclusions

To our knowledge, this is the first study that compares paired deep and shallow sites within RA patients. Our findings suggest that the aggregate subgingival microbiome is not significantly different between individuals with and without rheumatoid arthritis and that the probing depth primarily drives the differences in the overall profile of the subgingival microbiome. Compared to the substantial differences in subgingival microbiome typically seen between deep and shallow sites in the general non-RA population [64], our data suggest that these communities are more similar to each other in RA patients. These results raise the hypothesis that factors associated with the onset of RA may modulate the ecology of subgingival microbiome and its relationship to periodontal disease, the basis of which remains unknown but warrants further investigation. Additionally, we identified several OTUs, which were differentially abundant between RA patients and non-RA controls, and their role in RA and periodontal disease should be explored in future studies.

## Supplementary Information

**Additional file 1. Figure S1**. Differentially abundant OTUs between deep and shallow sites in non-RA controls. Differentially abundant taxa were identified using LEfSe and met the minimum LDA score of 2. OTUs enriched in deep subgingival sites are shown in red, and OTUs more abundant in shallow sites are shown in dark blue. OTU: Operational taxonomic unit.** Figure S2**: Differentially abundant metagenome functions between deep and shallow sites in non-RA controls. Metagenome functions were predicted using 16S rRNA data and PICRUSt. Differentially abundant gene functions were identified using LEfSe and met the minimum LDA score of 2. Gene functions enriched in deep sites are shown in red, whereas functions more abundant in shallow sites are shown in dark blue.

**Additional file 2. Table S1**. Association between RA disease severity and alpha diversity of the subgingival microbiome. Mixed linear models that included the RAPID3 score of RA disease severity and probe depth (shallow vs. deep) were created for each alpha diversity metric. Subject identity was included as a random effect. Model coefficient estimates, standard error (SE) of the estimates, and p values are shown. OTUs: Operational taxonomic units.

## Data Availability

The datasets (DOI: 10.17026/dans-zsb-wg2f) generated and/or analyzed during the current study are available in the Data Archiving and Networked Service (DANS) repository [10.17026/dans-zsb-wg2f].

## Abbreviations

RA: Rheumatoid arthritis
ACPAs: Autoantibodies to citrullinated proteins
PD: Probing depth
CAL: Clinical attachment level
BoP: Bleeding upon probing
