# Supplementary material for: Subgingival microbiome of deep and shallow periodontal sites in patients with rheumatoid arthritis: a pilot study
Source: BMC Oral Health. 2021 May 8;21:248. doi: 10.1186/s12903-021-01597-x (PMC8105973; doi:10.1186/s12903-021-01597-x)
Supplement: Supplementary file 1 — Additional file 1. Figure S1. Differentially abundant OTUs between deep and shallow sites in non-RA controls. Differentially abundant taxa were identified using LEfSe and met the minimum LDA score of 2. OTUs enriched in deep subgingival sites are shown in red, and OTUs more abundant in shallow sites are shown in dark blue. OTU: Operational taxonomic unit. Figure S2: Differentially abundant metagenome functions between deep and shallow sites in non-RA controls. Metagenome functions were predicted using 16S rRNA data and PICRUSt. Differentially abundant gene functions were identified using LEfSe and met the minimum LDA score of 2. Gene functions enriched in deep sites are shown in red, whereas functions more abundant in shallow sites are shown in dark blue. [file 12903_2021_1597_MOESM1_ESM.docx]

**Subgingival microbiome of deep and shallow periodontal sites**

**in patients with rheumatoid arthritis: A pilot study**

Ryanne Lehenaff, BS, MSc, Ryan Tamashiro, BS, Marcelle M. Nascimento, DDS, MS, PhD, Kyulim Lee, BS, Renita Jenkins, Joan Whitlock, BS, Eric C. Li, MS, Gurjit Sidhu, PhD, Susanne Anderson, MD, Ann Progulske-Fox, PhD, Michael R. Bubb, MD, Edward K.L. Chan, PhD, Gary P. Wang, MD, PhD


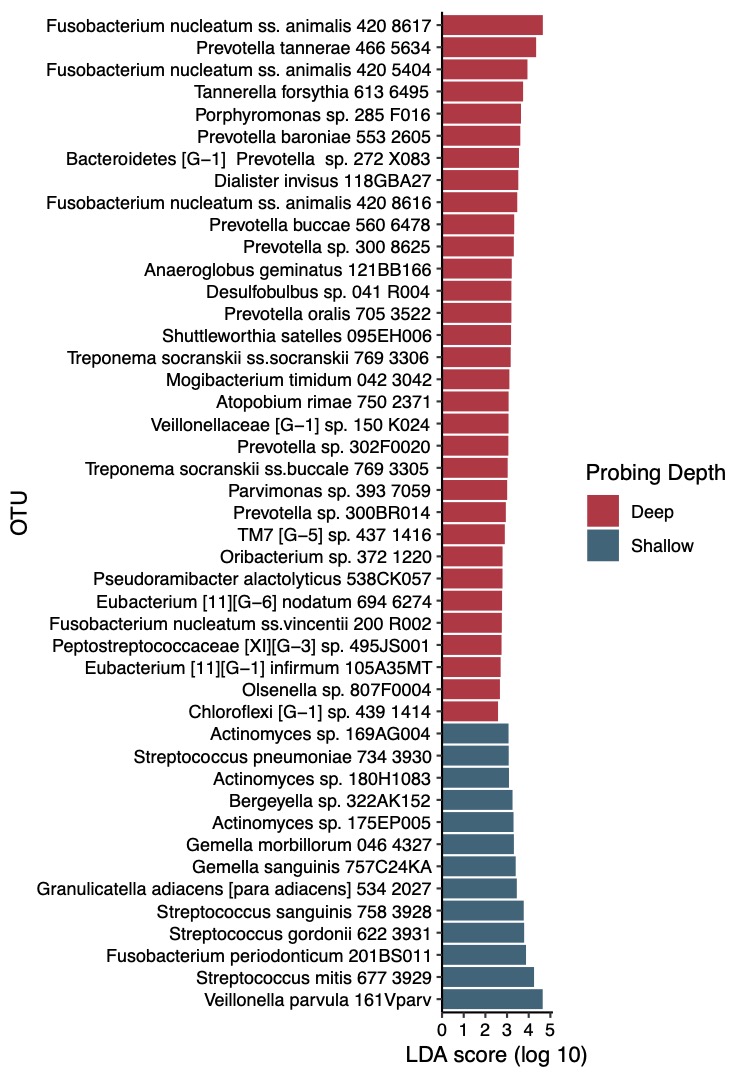


**Figure S1. Differentially abundant OTUs between deep and shallow sites in non-RA controls.** Differentially abundant taxa were identified using LEfSe and met the minimum LDA score of 2. OTUs enriched in deep subgingival sites are shown in red, and OTUs more abundant in shallow sites are shown in dark blue. OTU: Operational taxonomic unit.


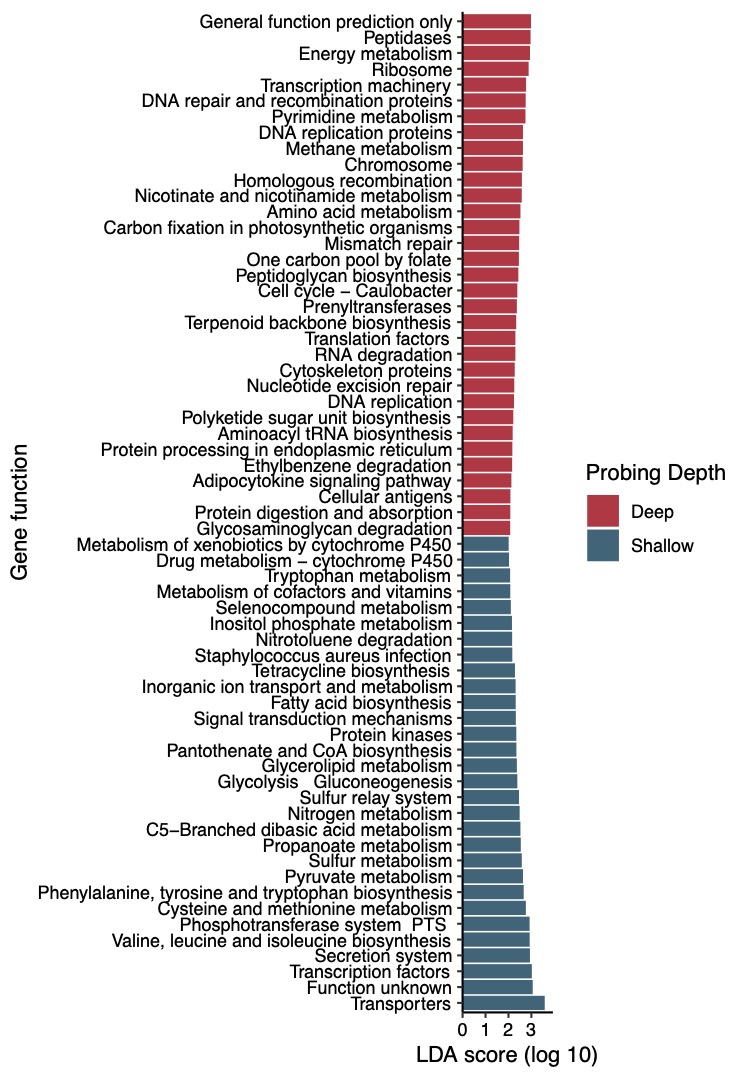


**Figure S2. Differentially abundant metagenome functions between deep and shallow sites in non-RA controls.** Metagenome functions were predicted using 16S rRNA data and PICRUSt. Differentially abundant gene functions were identified using LEfSe and met the minimum LDA score of 2. Gene functions enriched in deep sites are shown in red, whereas functions more abundant in shallow sites are shown in dark blue.
