# Supplementary material for: Subgingival microbiome of deep and shallow periodontal sites in patients with rheumatoid arthritis: a pilot study
Source: BMC Oral Health. 2021 May 8;21:248. doi: 10.1186/s12903-021-01597-x (PMC8105973; doi:10.1186/s12903-021-01597-x)
Supplement: Supplementary file 2 — Additional file 2. Table S1. Association between RA disease severity and alpha diversity of the subgingival microbiome. Mixed linear models that included the RAPID3 score of RA disease severity and probe depth (shallow vs. deep) were created for each alpha diversity metric. Subject identity was included as a random effect. Model coefficient estimates, standard error (SE) of the estimates, and p values are shown. OTUs: Operational taxonomic units. [file 12903_2021_1597_MOESM2_ESM.docx]

**Subgingival microbiome of deep and shallow periodontal sites**

**in patients with rheumatoid arthritis: A pilot study**

Ryanne Lehenaff, BS, MSc, Ryan Tamashiro, BS, Marcelle M. Nascimento, DDS, MS, PhD, Kyulim Lee, BS, Renita Jenkins, Joan Whitlock, BS, Eric C. Li, MS, Gurjit Sidhu, PhD, Susanne Anderson, MD, Ann Progulske-Fox, PhD, Michael R. Bubb, MD, Edward K.L. Chan, PhD, Gary P. Wang, MD, PhD

|  | Observed OTUs | Faith’s phylogenetic diversity | Shannon diversity |
| --- | --- | --- | --- |
| RAPID3 | 4.33 ± 2.57SE,  p = 0.139 | 0.18 ± 0.15SE,  p = 0.255 | 0.05 ± 0.06SE,  p = 0.417 |
| Probe depth  (ref: Shallow) | 7.55 ± 16.93SE,  p = 0.671 | 0.78 ± 0.73SE,  p = 0.329 | 0.42 ± 0.36SE,  p = 0.291 |

**Table S1.** **Association between RA disease severity and alpha diversity of the subgingival microbiome.** Mixed linear models that included the RAPID3 score of RA disease severity and probe depth (shallow vs. deep) were created for each alpha diversity metric. Subject identity was included as a random effect. Model coefficient estimates, standard error (SE) of the estimates, and p values are shown. OTUs: Operational taxonomic units.
